# Single-cell transcriptome and multi-omics integration reveal ferroptosis-driven immune microenvironment remodeling in knee osteoarthritis

**DOI:** 10.3389/fimmu.2025.1608378

**Published:** 2025-06-25

**Authors:** Yushun Wu, Jing Liu, Wenying Yu, Xiaoding Wang, Jian Li, Weiquan Zeng

**Affiliations:** ^1^ Department of Sports Medicine, The Second Affiliated Hospital of Fujian University of Traditional Chinese Medicine, Fuzhou, China; ^2^ Department of Pain Management, The Affiliated Hospital of Fujian University of Traditional Chinese Medicine, Fuzhou, China; ^3^ The First Clinical Medical College, Fujian University of Traditional Chinese Medicine, Fuzhou, China; ^4^ College of Computer and Network Space Security, Fujian Normal University, Fuzhou, China; ^5^ Department of Orthopedics, The Rehabilitation Hospital of Fujian University of Traditional Chinese Medicine, Fuzhou, China

**Keywords:** knee osteoarthritis, ferroptosis, single-cell transcriptomics, immune microenvironment, diagnostic biomarkers

## Abstract

**Background:**

Knee osteoarthritis (KOA) is a chronic inflammatory joint disorder marked by cartilage degradation and immune microenvironment dysregulation. While transcriptomic studies have identified key pathways in KOA, the interplay between ferroptosis (an iron-dependent cell death mechanism) and immune dysfunction at single-cell resolution remains unexplored. This study integrates single-cell and bulk transcriptomics to dissect ferroptosis-driven immune remodeling and identify diagnostic biomarkers in KOA.

**Methods:**

We analyzed scRNA-seq data (GSE255460, *n* = 11) and bulk RNA-seq cohorts (GSE114007: 20 KOA/18 controls; GSE246425: 8 KOA/4 controls). Single-cell data were processed via Seurat (QC: mitochondrial genes >3 MAD; normalization: LogNormalize; batch correction: Harmony) and annotated using CellMarker/PanglaoDB. CellChat decoded intercellular communication, SCENIC reconstructed transcriptional networks, and Monocle2 for pseudotime trajectory mapping. Immune infiltration (CIBERSORT) and a LASSO-SVM diagnostic model were validated by ROC curves. Functional enrichment (GSEA/GSVA) and immunometabolic profiling were performed.

**Results:**

Twelve chondrocyte clusters were identified, including ferroptosis-active homeostasis chondrocytes (HomC) (*p* < 0.01), which exhibited 491 DEGs linked to lipid peroxidation. HomC orchestrated synovitis via FGF signaling (ligand-receptor pairs: FGF1-FGFR1), amplifying ECM degradation and inflammatory cascades (CellChat). SCENIC revealed 10 HomC-specific regulons (e.g., *SREBF1*, *YY1*) driving matrix metalloproteinase activation. A 7-gene diagnostic panel (*IFT88*, *MIEF2*, *ABCC10*, etc.) achieved AUC = 1.0 (training) and 0.78 (validation). Immune profiling showed reduced resting mast cells (*p* = 0.003) and monocytes (*p* = 0.02), with *ABCC10* correlating negatively with CD8+ T cells (*r* = -0.65) and M1 macrophages. GSEA/GSVA implicated HIF-1, NF-κB, and oxidative phosphorylation pathways in KOA progression. Pseudotime analysis revealed fibrotic transitions (*COL1A1*↑, *TNC*↑) in late-stage KOA cells.

**Conclusion:**

This study establishes ferroptosis as one of the key drivers immune-metabolic dysfunction in KOA, with HomC acting as a hub for FGF-mediated synovitis and ECM remodeling. The diagnostic model and regulon network (*SREBF1/YY1*) offer translational tools for early detection, while impaired mast cell homeostasis highlights novel immunotherapeutic targets. Our findings bridge ferroptosis, immune dysregulation, and metabolic stress, advancing precision strategies for KOA management.

## 1 Introduction

Knee osteoarthritis (KOA) is a chronic inflammatory disease of the joint, characterized by degenerative changes in cartilage and dysregulation of the immune microenvironment (1, 2). KOA has a major adverse impact on patients’ quality of life and poses a considerable economic burden on healthcare systems globally (3, 4). Current management strategies of KOA comprise medications across various pharmacological classes, physical interventions, and surgical procedures, which often show limited treatment efficacy with varying adverse effects (5, 6). This exemplifies the urgent need to develop new treatment approaches for KOA.

The recent use of transcriptomic approaches have generated valuable information regarding gene expression profiles in KOA, noting inflammatory pathways and immune cell infiltration as contributors to disease progression (7, 8). However, these studies mostly examine bulk tissue samples which mask the cellular heterogeneity in the joint and do not illustrate the complex cell-specific interactions that drive immune remodeling in KOA (9, 10). At a cellular level, a number of types of immune cells, such as macrophages, mast cells, and T-lymphocytes, may interact in the synovial microenvironment to drive inflammation and matrix degradation (11–13).

Ferroptosis, a form of iron-dependent regulated cell death, has emerged as a critical mechanism in various diseases, including osteoarthritis (14, 15). This process is characterized by lipid peroxidation and oxidative stress, both of which are central to the pathophysiology of KOA (16, 17). Despite its significance, the role of ferroptosis in immune microenvironment remodeling and cartilage degradation in KOA has not been explored in detail, particularly at the single-cell level.

To address this knowledge gap, we combine single-cell RNA sequencing (scRNA-seq) and bulk transcriptomic data to provide a multi-faceted overview of the immune microenvironment in KOA. This multi-omics approach allows us to examine the interplay of ferroptosis and immune dysregulation in KOA. The distinguishing feature of scRNA-seq is its ability to demonstrate cell heterogeneity and the subpopulations of immune cells that may be involved in the pathogenic processes to an increasing degree of resolution (18, 19). This will allow for a greater understanding of how ferroptosis impacts immune remodeling and contributes to disease progression (20).

The primary aim of this study is to identify biomarkers associated with ferroptosis driven immune microenvironment remodeling in KOA. These biomarkers have the potential to be used as early diagnostic tools and as targets for therapeutic intervention for new treatment approaches (21). The hope is that an enhanced understanding of the molecular mechanisms occurring in KOA will provide new insights into disease pathogenesis, as well as provide new therapeutic avenues for the development of more efficacious treatments for KOA.

## 2 Methods

### 2.1 Data acquisition

GEO database (https://www.ncbi.nlm.nih.gov/geo/info/datasets.html) the full name of GENE EXPRESSION OMNIBUS, is by the us national center for biotechnology information (NCBI database creation and maintenance of GENE EXPRESSION. To ensure clarity, we explicitly detail the origin and characteristics of each dataset used in this study. GSE255460 comprises single-cell RNA sequencing data from human knee articular cartilage tissue of 11 patients with KOA. The annotation platform was GPL24676, and batch effect correction was performed using the Harmony algorithm. GSE114007 consists of bulk mRNA expression data from knee articular cartilage tissue, including 20 samples from OA patients and 18 samples from healthy controls, and served as the model training set. GSE246425 provides mRNA expression profiles of *in vitro* cultured human chondrocytes, incorporating samples from both OA patients and healthy donors; chondrocytes were cultured to replicative senescence (Hayflick limit) to establish a senescent cell model. This dataset, also annotated with GPL24676, was used as a validation cohort and comprised 8 OA samples and 4 control samples. Notably, both GSE255460 and GSE114007 were sourced from knee cartilage tissue, whereas GSE246425 was derived from isolated chondrocytes cultured *in vitro*. For all differential analyses involving two groups, the Wilcoxon test was employed. Since only two groups (control vs. OA) were compared, ANOVA was not applied. Detailed inclusion and exclusion criteria, OA severity grading, sample collection, and single-cell isolation methods for GSE255460 are available in the original publication. All data were derived from publicly available datasets with original ethical approvals documented in the primary studies.

### 2.2 Single cell data quality control

The expression profile was first read through the Seurat packet, in which we filtered the cells according to the total UMI per cell, the number of genes expressed (22), and the proportion of mitochondrial expression per cell. The proportion of mitochondrial gene expression refers to the percentage of the total expression of mitochondrial genes in the total expression of all genes. Cells with a high proportion of mitochondrial gene expression and a low amount of RNA expression are entering the death process. For rigorous quality control, we employed the median absolute deviation (MAD) method. Variables (such as UMI counts, gene numbers, or mitochondrial content) exceeding 3 MADs from the median were identified as outliers and excluded from downstream analyses. Multiple-testing correction in differential expression analysis was performed using the Benjamini-Hochberg method to control the false discovery rate (FDR).

### 2.3 Single cell data dimensionality reduction clustering and cell annotation

We adopt the globally standardized LogNormalize method, by multiplying a coefficient s0, the total expression of each cell is adjusted to 10000, and then logarithm is taken for standardization. Cell cycle score was calculated using CellCycleScoring. FindVariableFeatures Looks for highly variable genes; We used ScaleData function to remove gene expression fluctuations caused by different mitochondrial gene expression proportion, ribosomal gene expression proportion and cell cycle. The expression matrix was reduced linearly by RunPCA, and principal components were selected for subsequent analysis. The batch effect was removed by Harmony, and the non-linear dimensionality reduction was performed by RunUMAP Unified Manifold Approximation and Projection (UMAP). By searching CellMarker and PanglaoDB database and literature mainly (23, 24), supplemented by automated annotation by SingleR software, cell types existing in corresponding tissues and corresponding marker genes were searched for cell annotation.

### 2.4 Ligand receptor interaction analysis (CellChat)

CellChat is a tool that enables quantitative inference and analysis of intercellular communication networks from single-cell data (25). CellChat uses network analysis and pattern recognition methods to predict the major signal inputs and outputs of cells and how these cells and signals coordinate functions. In this analysis, we used standardized single-cell expression profiles as input data, and cell subtypes obtained from single-cell analysis were cell information. Cell-related interactions were analyzed, and weights and counts of inter-cell interactions were used to quantify the closeness of interactions, so as to observe the activity and influence of each type of cell in disease.

### 2.5 SCENIC analysis

SCENIC(single-cell regulatory network inference and clustering) is a method for calculating gene regulatory network reconstruction and cell state identification based on co-expression and motif analysis of single-cell transcriptome data (26). It first identified the set of genes co-expressed with transcription factors through GENIE3, then carried out motif enrichment analysis for each co-expression module, retained significantly enriched motifs, and TF annotation of motifs using the database, and the annotation results were rated as high and low confidence. TF for direct database annotation and homologous gene inference are high confidence results, TF for motif sequence similarity annotation is low confidence results. The genes in the co-expression module are scored with the retained motif, the genes with significantly high scores are identified (understood as the motif is very close to the TSS of these genes), the genes with low scores in the co-expression module are deleted, and the remaining gene set is called regulon. Each regulon is a transcription factor and the set of genes that directly regulate its target genes, and SCENIC’s next job is to score each regulon’s activity in individual cells. The score is based on the expression value of the gene, the higher the score represents the higher the degree of activation of the gene set, and the resulting activity matrix can identify the cell type and state.

### 2.6 Construction of prediction model

Lasso regression was used to further construct the prediction correlation model (27). After incorporating the expression value of each specific gene, a risk score formula for each patient was constructed, weighted by its estimated regression coefficient in lasso regression analysis. The score for each patient was calculated according to the risk score formula, and the ROC curve was used to study the accuracy of the model prediction. At the same time, SVM algorithm was used for feature selection of disease diagnostic markers (28). Svm-rfe is a machine learning method based on support vector machines, which seeks the best variables by deleting the feature vectors generated by SVM, and builds a support vector machine model through the “e1071” software package to further identify the diagnostic value of these biomarkers for diseases.

### 2.7 Immune infiltration

CIBERSORT method is a widely used method to evaluate immune cell types in microenvironment (29). Based on the principle of support vector regression, the expression matrix of immune cell subtypes was deconvolution analyzed. It contains 547 biomarkers that distinguish 22 human immune cell phenotypes, including T cells, B cells, plasma cells, and myeloid cell subsets. In this study, CIBERSORT algorithm was used to analyze patient data, which was used to infer the relative proportion of 22 kinds of immune infiltrating cells, and correlation analysis was conducted for gene expression and immune cell content.

### 2.8 GSEA analysis

Gene Set Enrichment Analysis (GSEA) was conducted to assess the differences in pathway enrichment among groups (30). In accordance with the expression levels of the specific genes in the samples, samples were assigned to either a high or low expression group and differences between groups were further examined through the GSEA process. The background gene sets were annotation gene sets of version 7.0 downloaded from the MsigDB database and were considered as annotation gene sets of the subtype type pathways. The process involved differential expression analysis of the pathways between the groups. All significantly enriched gene sets, adjusted p value <0.05, were ranked based upon the consistency scores. GSEA is frequently used to examine the close association of disease classification with biological process significance.

### 2.9 GSVA analysis

Gene set variation analysis (GSVA) is an unsupervised, non-parametric method for assessing the enrichment of gene sets from transcriptome data (31). GSVA examines the gene set of interest in a cumulative score to convert the gene level change into pathway level change, and then attempts to make biological function assertions about the sample. In this study, gene sets were downloaded from Molecular Signatures Database, and we computed comprehensive scores for each of the gene sets using the GSVA algorithm to assess potential biological functional changes in the different samples.

### 2.10 Quasi-temporal analysis

Studies at the single-cell level have made it possible to describe transcriptional regulation of complex physiological processes and highly heterogeneous cell populations. These studies contribute to the discovery of genes that recognize specific cell subtypes, genes that mark intermediate states of biological processes, and genes that transition states between two different cell fates. In many single-cell studies, individual cells perform the gene expression process in an unsynchronized manner, and each cell is a moment of the transcription process being studied. Monocle introduced the strategy of sequencing individual cells within pseudotime (32), using the non-synchronous processes of individual cells to place them on a trajectory corresponding to biological processes such as cell differentiation.

### 2.11 Statistical analysis

All statistical analyses were conducted using R language (version 4.3.0), with p<0.05 being statistically significant.

## 3 Results

### 3.1 Integration of single-cell data and identification of cellular subpopulations

**Figure 1:**
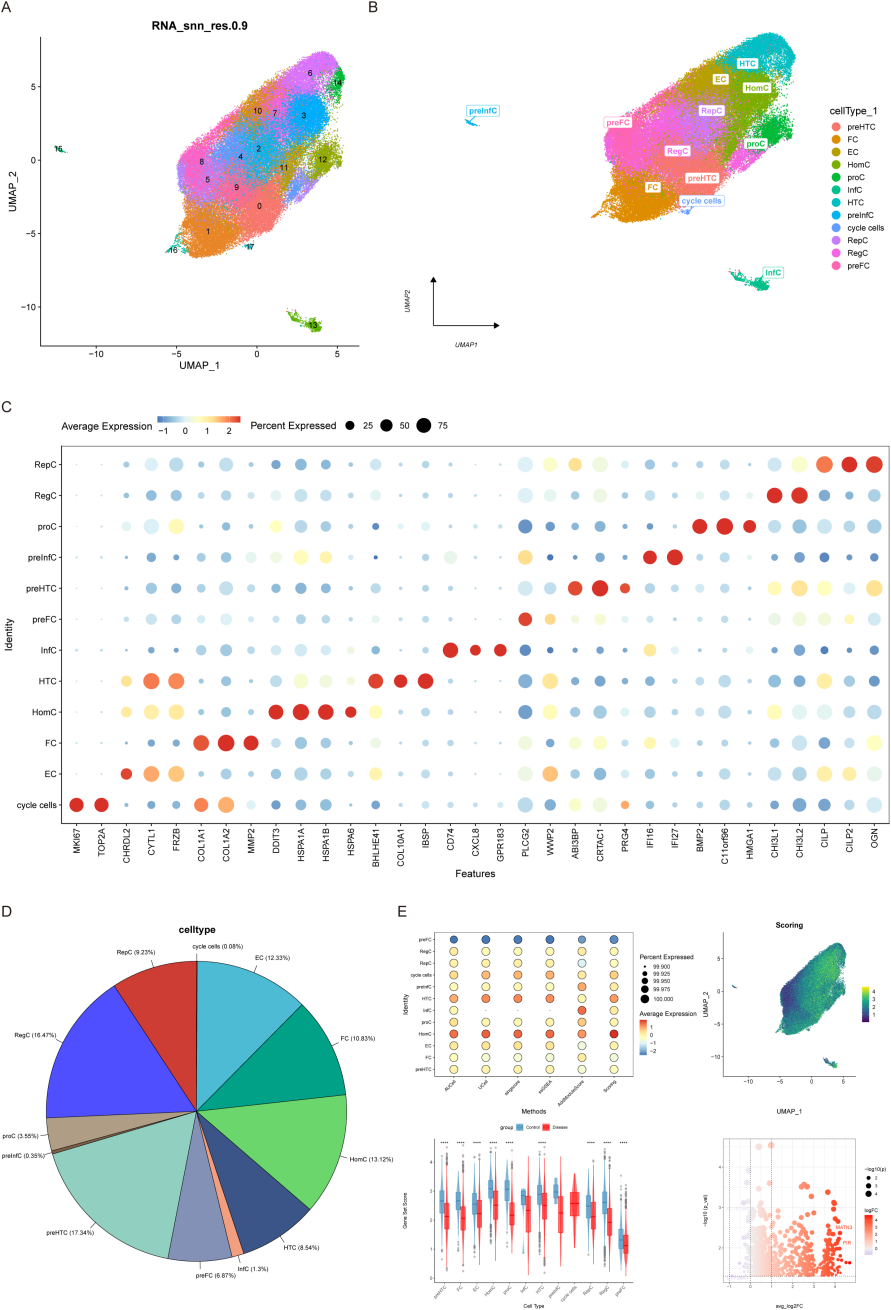
Cellular annotation and differences in ferroptosis scores. **(A)** Cells were grouped into 18 clusters using UMAP algorithm based on important components available from PCA. **(B)** Annotation of the 18 clusters, categorized into 12 cell types: preHTC (prehypertrophic chondrocytes), FC (fibrocartilage chondrocytes), EC (effector chondrocytes), HomC (homeostasis chondrocytes), proC (proliferation chondrocytes), InfC (inflammatory chondrocytes), HTC (hypertrophic chondrocytes), preInfC (pre-inflammatory chondrocytes), RepC (reparative chondrocytes), RegC (regulator chondrocytes), preFC (prefibrocartilage chondrocytes), and cycle cells. **(C)** Bubble plot of 12 cell types and their corresponding cell markers in the Doplot. **(D)** Pie chart illustrating the proportion of 12 cell types.**(E)** Differential expression of ferroptosis scores across the 12 cell types.

Considering the data quality of multiple samples, the captured outliers of the cells will be filtered. In the end, a total of 74,182 cells were retained, and the violin diagram and scatter diagram after quality control (**Supplementary Figures 1A**, **Figure 1B**) were obtained. We then analyzed 2000 highly variable genes and showed the 10 genes with the highest standard deviation among them (**Supplementary Figure 1C**). Subsequently, the data were standardized, homogenized, PCA and harmony analyzed and processed successively (**Supplementary Figures 1D–F**). UMAP plots before and after Harmony correction in the **Supplementary Figures 1E, F**, which demonstrate that cell clustering by batch has been effectively minimized. After dimensionality reduction by unified manifold approximation and projection (UMAP), a total of 18 subgroups are obtained (**Figure 1A**). The study further annotated each cell subtype. In total, 12 cell types were identified and annotated: preHTC (prehypertrophic chondrocytes), FC (fibrocartilage chondrocytes), EC (effector) chondrocytes), HomC (homeostasis chondrocytes), proC (proliferation chondrocytes), InfC (inflammatory) chondrocytes), HTC (hypertrophic chondrocytes), preInfC (pre-inflammatory chondrocytes), RepC (reparative) There were 12 types of cells, namely chondrocytes, RegC (regulator chondrocytes), preFC (prefibrocartilage chondrocytes) and cycle cells (**Figure 1B**). The classic marker genes for each of the 12 cell types are shown in a bubble plot (**Figure 1C**), and the proportions of each cell type are illustrated in **Figure 1D**.

### 3.2 Ferroptosis mechanisms and identification of key cell types

Then we gain the iron from iron death database (http://www.zhounan.org/ferrdb) death related genes, a total of 484 iron death related genes, AUCell, UCell, singscore, ssGSEA and Add algorithms were used to evaluate iron death at the level of scRNA-seq, and the scores of the above algorithms were averaged to evaluate the expression of iron death in different cell types. According to the results, the cell subsets with a small number of cells were eliminated. In the control group and the disease group, the expression of iron death was significantly different in HomC and the total quantified score was the highest in the two groups, so HomC was taken as the key cell (**Figure 1E**). Subsequently, we extracted HomC and used pseudo bulk to analyze the difference between the high and low HomC scores according to the median value of its iron death quantization score. The differential gene screening conditions were p-value < 0.05 and |logFC| > 1. A total of 491 differential genes (Candidate_Genes.txt) were obtained.

### 3.3 Intercellular communication analysis reveals critical pathways

**Figure 2:**
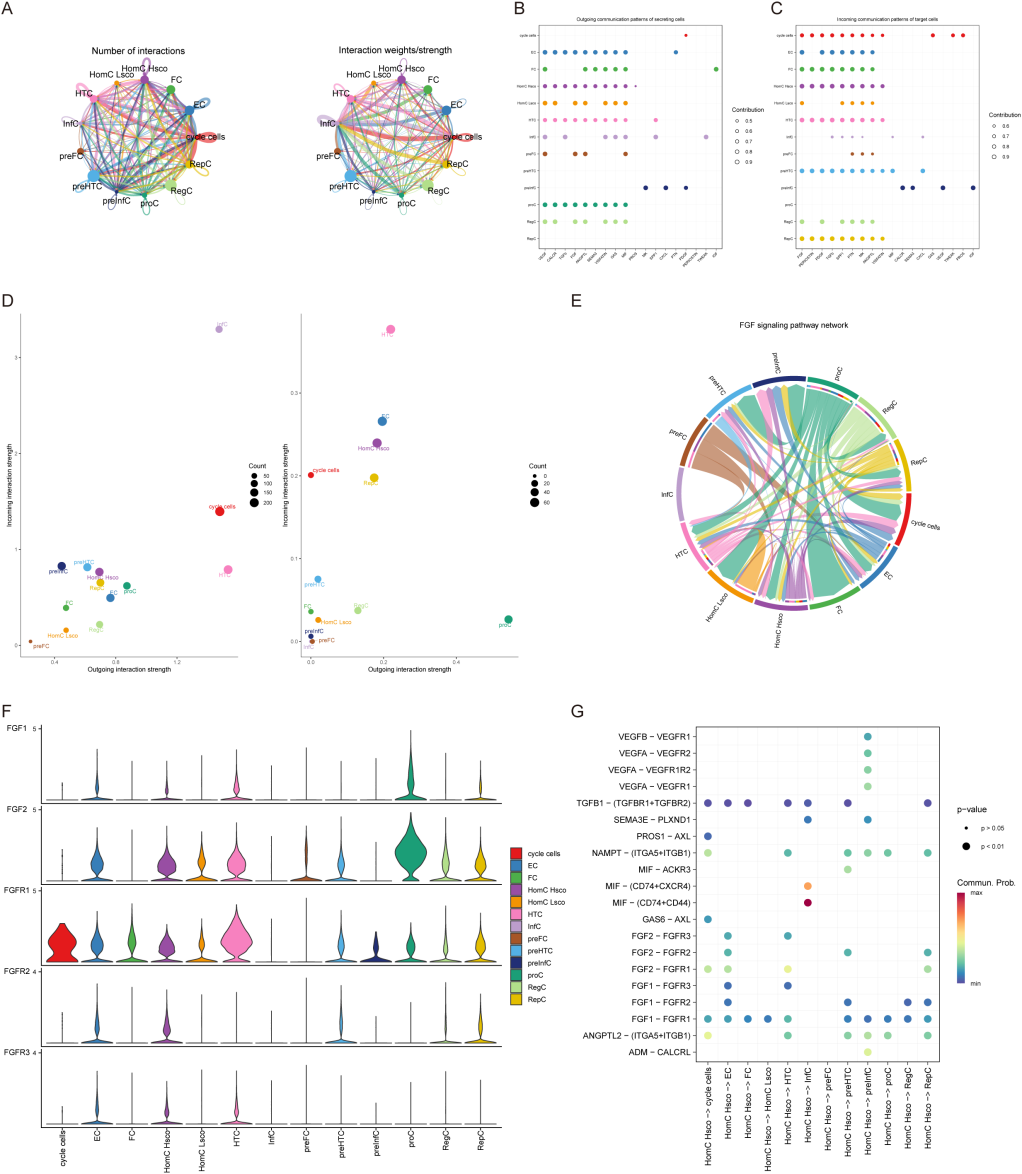
Cell-cell interactions. **(A)** The number and strength of interactions between cell subpopulations. **(B, C)** Cell communication through signaling pathways, with FGF signaling as the major ligand-receptor docking pathway. **(D)** Signal intensity from THomC is higher than other cells. **(E)** Interaction network between cells and the FGF signaling pathway in chord diagram format. **(F)** Violin plot of the expression level of FGF signaling pathway in cells. **(G)** Bubble plot showing receptor-ligand interactions between cells.

First, HomC was divided into high-low scores according to quantitative scores. Then, we analyzed the ligand-receptor relationship of feature in single cell expression profile using the software package cellchat. We found complex interaction pairs among these cell subtypes (**Figure 2A**). Then, we calculated the signal receiving and sending intensity of all cells for all signaling pathways (**Figures 2B, C**), and found that for HomC, the signal receiving and sending intensity of FGF signaling pathway was higher, pretending to be a critical signaling pathway analysis. Our analysis highlighted the significance of FGF signaling in mediating intercellular communication between HomC and synovial fibroblasts. Scatterplot analysis demonstrated robust FGF-mediated interactions between these cell types (**Figure 2D**). Chord diagram analysis further identified FGF1-FGFR1 as the most active ligand-receptor pair (**Figure 2E**), a finding that was corroborated by significantly elevated FGF1 expression in HomC (P < 0.001, **Figure 2F**). Functionally, HomC-derived FGF1 was found to promote the expression of MMP-13 and ADAMTS5 in synovial fibroblasts (**Figure 2G**), implicating this signaling axis in the degradation of the extracellular matrix (ECM).

### 3.4 Transcriptional regulatory network reconstruction identifies core regulators

**Figure 3:**
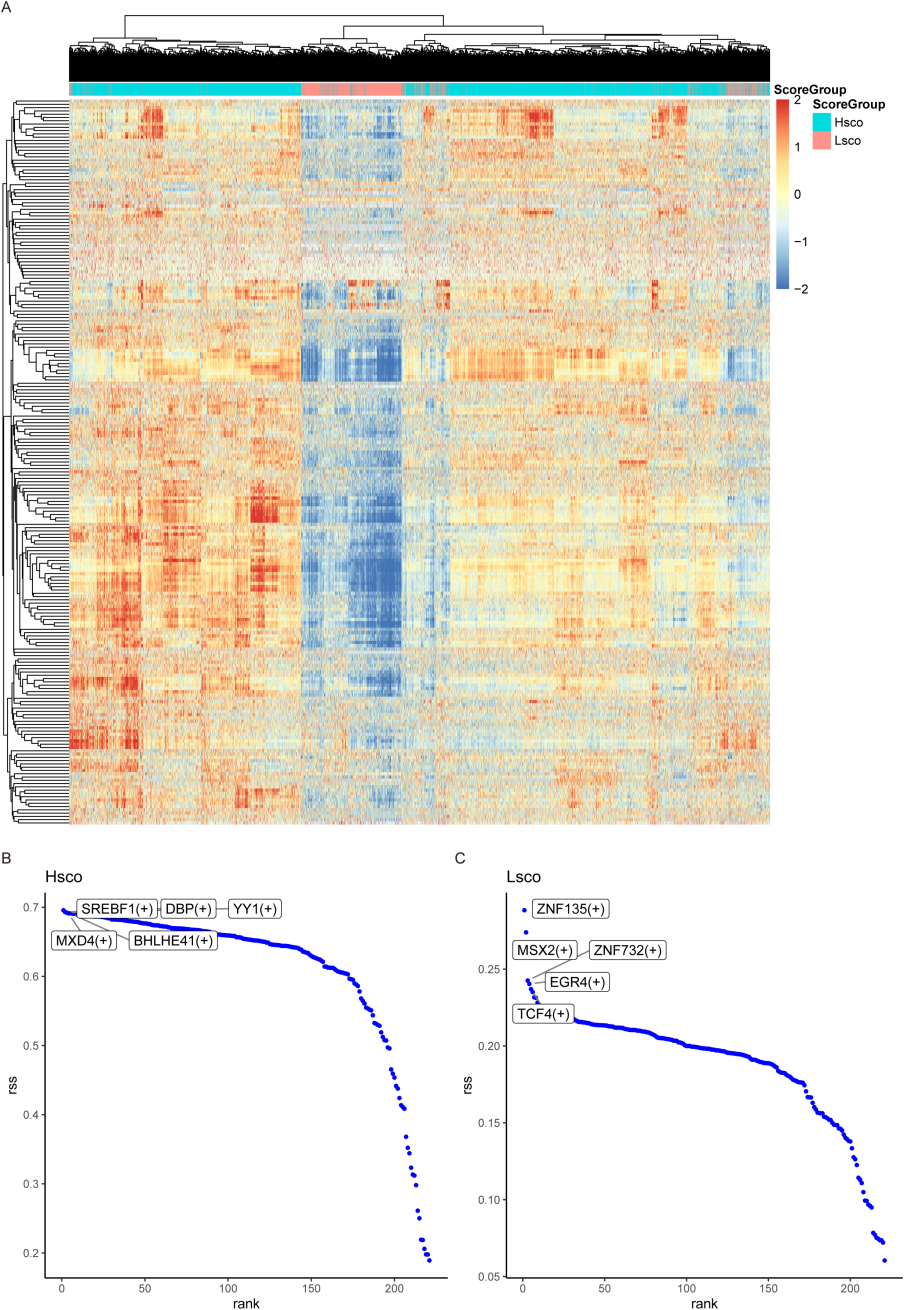
SCENIC analysis. **(A)** Heatmap displaying the regulon activity scores of each cell in the HomC subpopulation. SCENIC analysis identified all regulons in this subpopulation, and their activity distribution is visualized. **(B, C)** Scatter plots showing the specificity ranking of transcription factors in high-score and low-score groups.

We selected HomC for SCENIC analysis and output all regulatory units in this subpopulation, and we drew a heat map to visualize the regulon activity score for each cell (**Figure 3A**). We then show the relationship between a gene’s Rank and its residual sum of squares (RSS), which reflects the relative importance of each gene in the network. The results showed that SREBF1, DBP, YY1, MXD4, BHLHE41, ZNF135, MSX2, ZNF732, EGR4, TCF4 and other genes ranked first, and the regulator with higher RSS value may be specifically related to this cell population. Through RSS scores, we identified these 10 regulons as key regulatory elements in KOA (**Figures 3B, C**).

### 3.5 Multi-omics feature integration and construction of the diagnostic model

**Figure 4:**
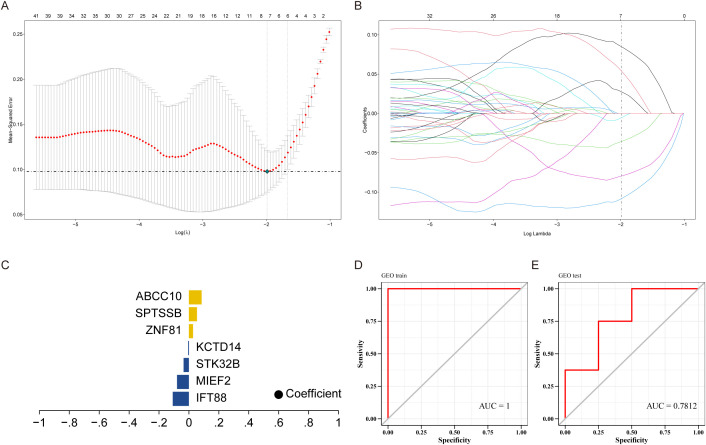
Construction of predictive models. **(A)** LASSO coefficient distribution of prognostic genes and gene combinations at the minimal lambda value. **(B)** Ten-fold cross-validation of the LASSO model to select the optimal lambda value. **(C)** Lasso coefficients for selected genes. **(D, E)** ROC curves for training and validation sets.

We downloaded the dataset of GSE114007 related to KOA from GEO database as the training set, and used the dataset GSE246425 as the validation set, and performed feature screening on 491 differential genes by Lasso regression. The results showed that Lasso regression consensus identified 7 genes as characteristic genes, as key genes for subsequent research, and built a prediction model (**Figures 4A–C**). The model formula is as follows: RiskScore = IFT88 (0.1082) + MIEF2 (0.0795) + STK32B (0.0356) + KCTD14 (0.0053)+ZNF81×0.0274+SPTSSB×0.0538+ABCC10×0.0848. The results showed that the prediction model constructed by 7 genes had better diagnostic efficiency, and the area under the AUC curve was 1 (**Figure 4D**). We further verified the diagnostic model against external data sets, and the results showed that the model had strong stability, and the area under the AUC curve of the verification set was 0.7812 (**Figure 4E**).

### 3.6 Correlation of model genes with the immune microenvironment

**Figure 5:**
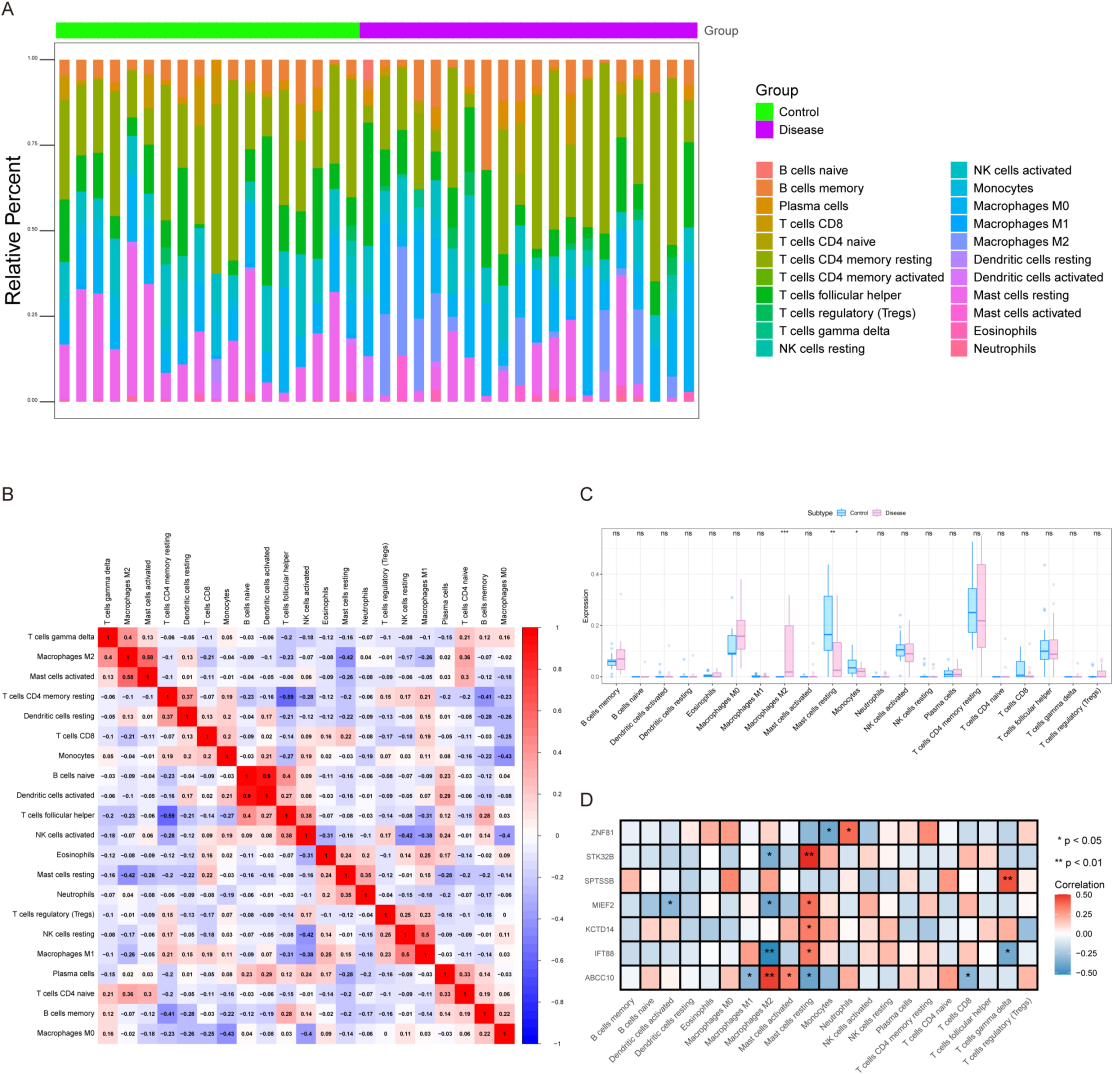
Immune infiltration analysis. **(A)** Relative percentages of immune cell subpopulations. **(B)** Correlation of immune cells, with blue representing negative correlation and red representing positive correlation. **(C)** Differences in immune cell content between control and disease samples. **(D)** Correlation between key genes and immune cells. ns, not significant (p > 0.05); *p < 0.05; **p < 0.01; ***p < 0.001.

**Figure 6:**
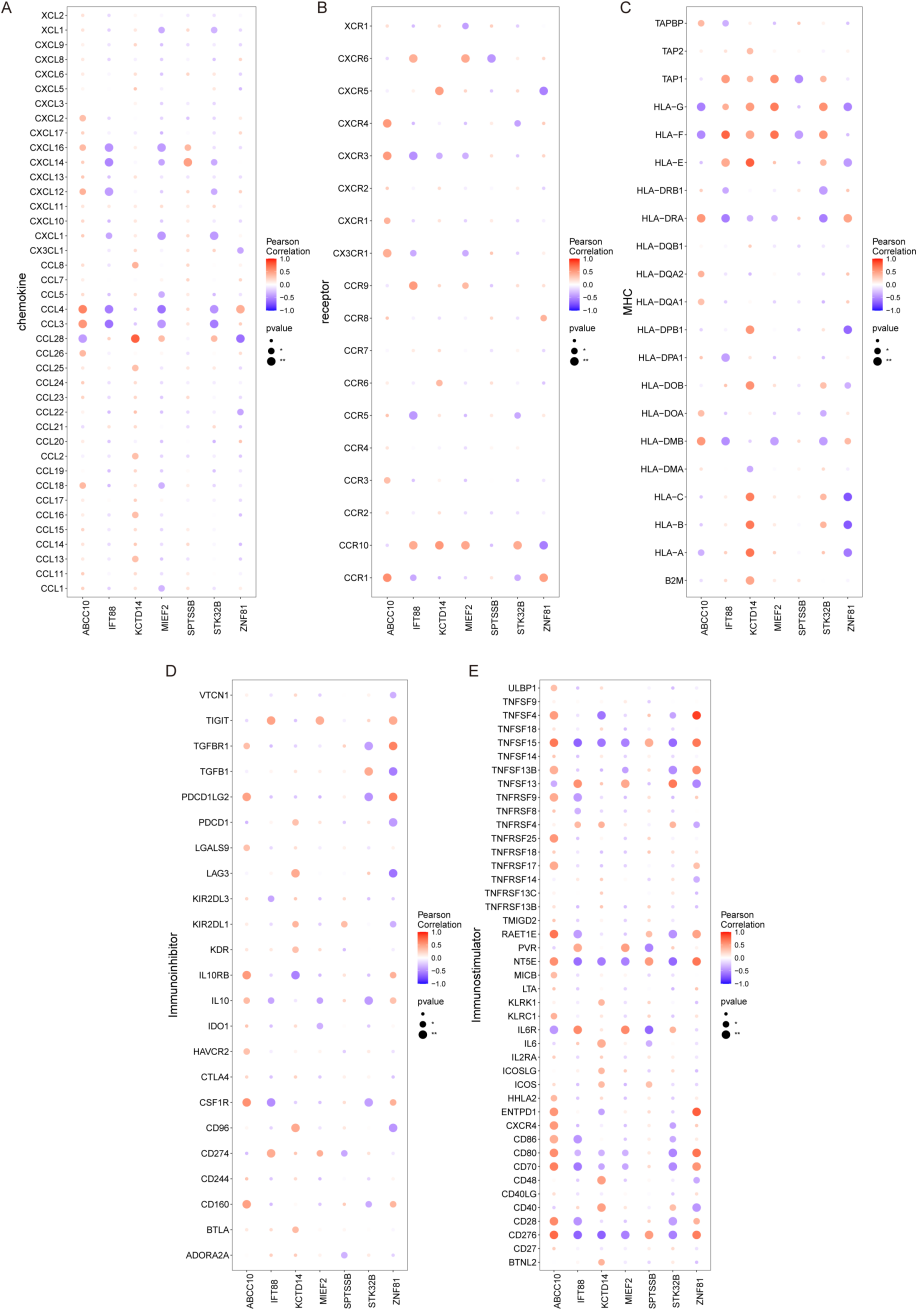
Relationship between key genes and immune factors. **(A–E)** Correlation of key genes with chemokines, immunoinhibitors, immunostimulators, MHC, and receptors.

The microenvironment is mainly composed of fibroblasts, immune cells, extracellular matrix, various growth factors, inflammatory factors and special physicochemical characteristics, which significantly affect the diagnosis, survival outcome and clinical treatment sensitivity of diseases. We showed the level distribution of immune infiltrations and the correlation of immune cells in different forms (**Figures 5A, B**). Compared with the control group, the levels of mast cells resting and monocytes in the samples from the disease group were significantly lower (**Figure 5C**). We further explored the relationship between key genes and immune cells, and found that IFT88 was significantly positively correlated with mast cells resting and negatively correlated with T cells gamma delta and macrophages M2. MIEF2 was positively correlated with mast cells resting and negatively correlated with Macrophages M2 and Dendritic cells activated. STK32B was positively correlated with mast cells resting and negatively correlated with Macrophages M2. KCTD14 was positively correlated with mast cells resting. ZNF81 was significantly positively correlated with Neutrophils, and negatively correlated with monocytes. SPTSSB was positively correlated with T cells gamma delta. ABCC10 is positively correlated with Macrophages M2 and mast cells activated, and negatively correlated with T cells CD8, Macrophages M1 and mast cells resting (**Figure 5D**). In addition, we analyzed the associations between key genes and different immune factors, including immunosuppressors, immunostimulators, chemokines, and receptors. These analyses suggest that key genes are closely related to the level of immune cell infiltration and play an important role in the immune microenvironment (**Figures 6A–E**).

We used AUCell to quantitatively score genes related to immune metabolism in single cells, and used bubble map to show the differences in the activity of key genes in immune metabolism related pathways. The results showed that SPTSSB, STK32B, ABCC10, ZNF81, KCTD14, IFT88 and MIEF2 were coagulation, mtorc1_signaling, unfolded_protein_response, myc_targets_v1, oxidative_phosphorylation and other pathways have higher activity (**Supplementary Figure 2**).

### 3.7 Trajectory evolution and functional mechanism validation

**Figure 7:**
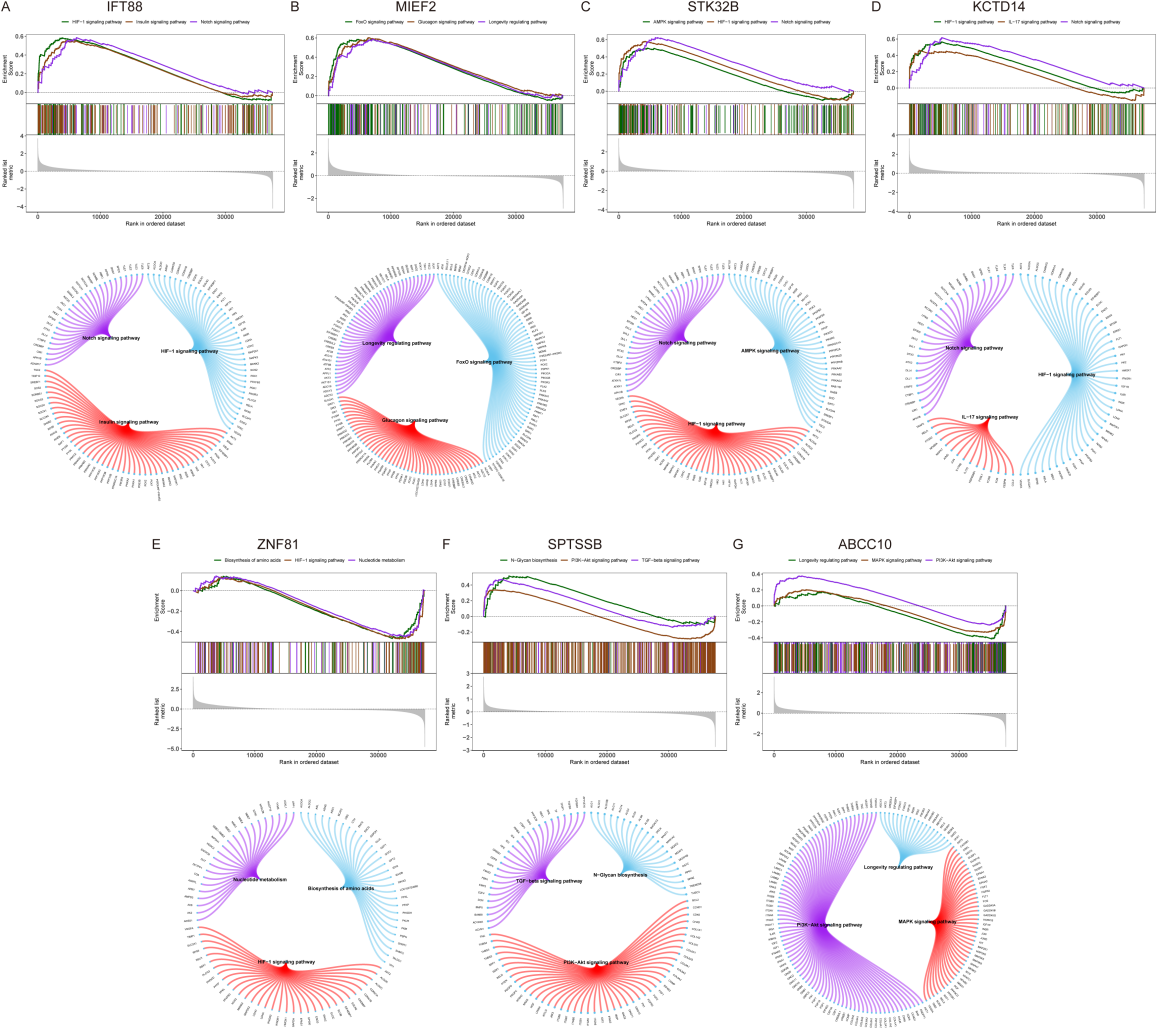
GSEA analysis of key genes. **(A–G)** KEGG signaling pathways involving key genes, including pathway regulation and associated genes.

Next, we investigate the specific signaling pathways involved in key genes and explore the underlying molecular mechanisms by which key genes influence disease progression. GSEA results showed that IFT88 was enriched in HIF-1 signaling pathway, Insulin signaling pathway, Notch signaling pathway and other signaling pathways (**Figure 7A**). MIEF2 is enriched in FoxO signaling pathway, Glucagon signaling pathway, Longevity regulating pathway and other signaling pathways (**Figure 7B**). STK32B is enriched in HIF-1 signaling pathway, Notch signaling pathway, AMPK signaling pathway and other signaling pathways (**Figure 7C**). KCTD14 is enriched in HIF-1 signaling pathway, Notch signaling pathway, IL-17 signaling pathway and other signaling pathways (**Figure 7D**). ZNF81 is enriched in HIF-1 signaling pathway, Biosynthesis of amino acids, Nucleotide metabolism and other signaling pathways (**Figure 7E**). SPTSSB is enriched in TGF-beta signaling pathway, PI3K-Akt signaling pathway, N-Glycan biosynthesis and other signaling pathways (**Figure 7F**). ABCC10 is enriched in PI3K-Akt signaling pathway, MAPK signaling pathway, Longevity regulating pathway and other signaling pathways (**Figure 7G**).

**Figure 8:**
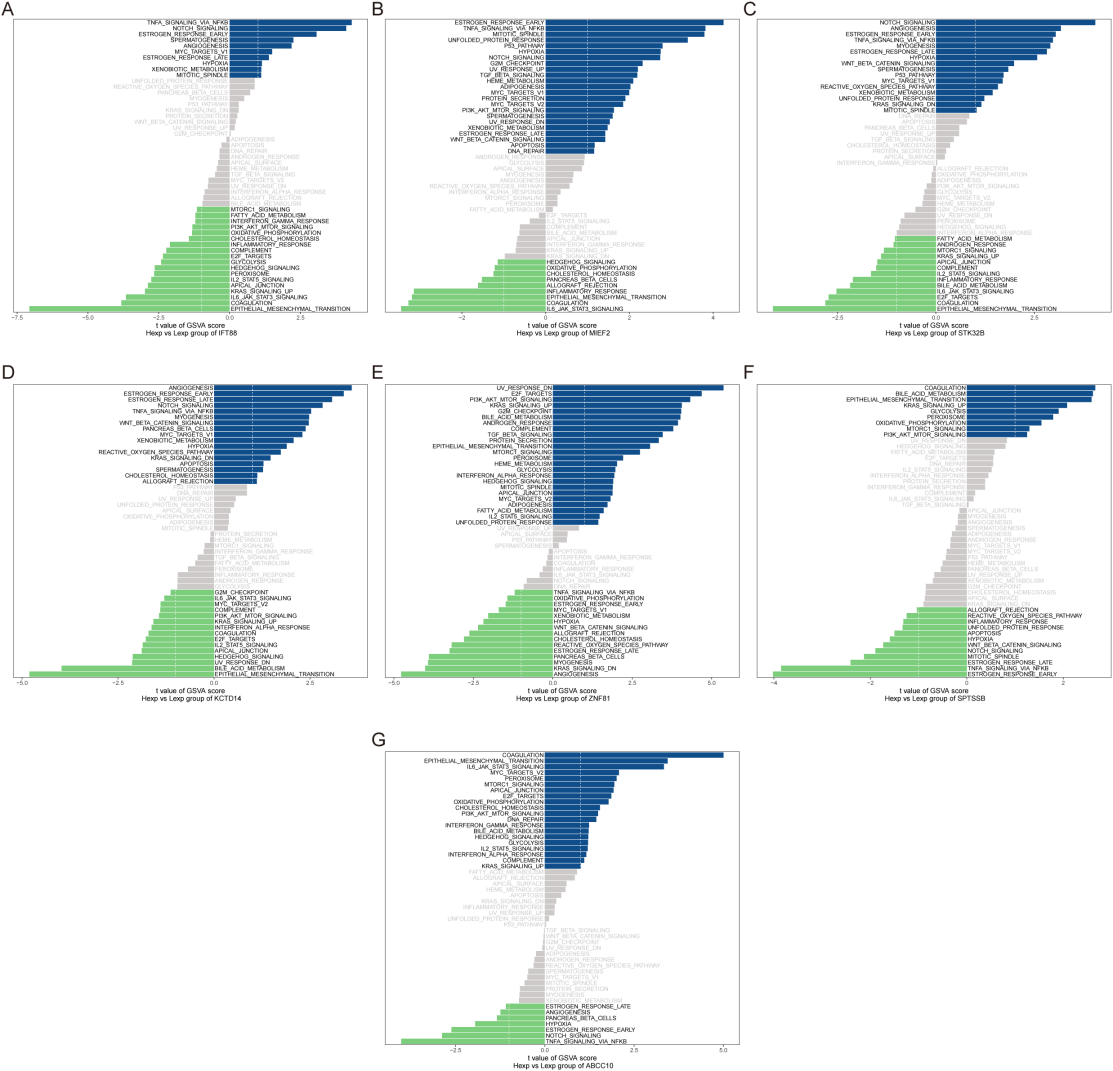
GSVA analysis of key genes. **(A–G)** GSVA analysis for key genes, with blue representing high-expression genes involved in signaling pathways and green representing low-expression genes. The hallmark gene set is used as the background.

GSVA analysis showed that IFT88 was enriched in TNFA_SIGNALING_VIA_NFKB and NOTCH_SIGNALING pathways (**Figure 8A**). MIEF2 is enriched in ESTROGEN_RESPONSE_EARLY, TNFA_SIGNALING_VIA_NFKB and other signaling pathways (**Figure 8B**). STK32B is enriched in NOTCH_SIGNALING, ANGIOGENESIS and other signaling pathways (**Figure 8C**). KCTD14 is enriched in ANGIOGENESIS, ESTROGEN_RESPONSE_EARLY and other signaling pathways (**Figure 8D**). ZNF81 is enriched in signal pathways such as UV_RESPONSE_DN and E2F_TARGETS (**Figure 8E**). SPTSSB is enriched in signal pathways COAGULATION, BILE_ACID_METABOLISM, etc. (**Figure 8F**). ABCC10 is enriched in signal pathways COAGULATION and EPITHELIAL_MESENCHYMAL_TRANSITION (**Figure 8G**). This suggests that key genes may influence disease progression through these pathways.

**Figure 9:**
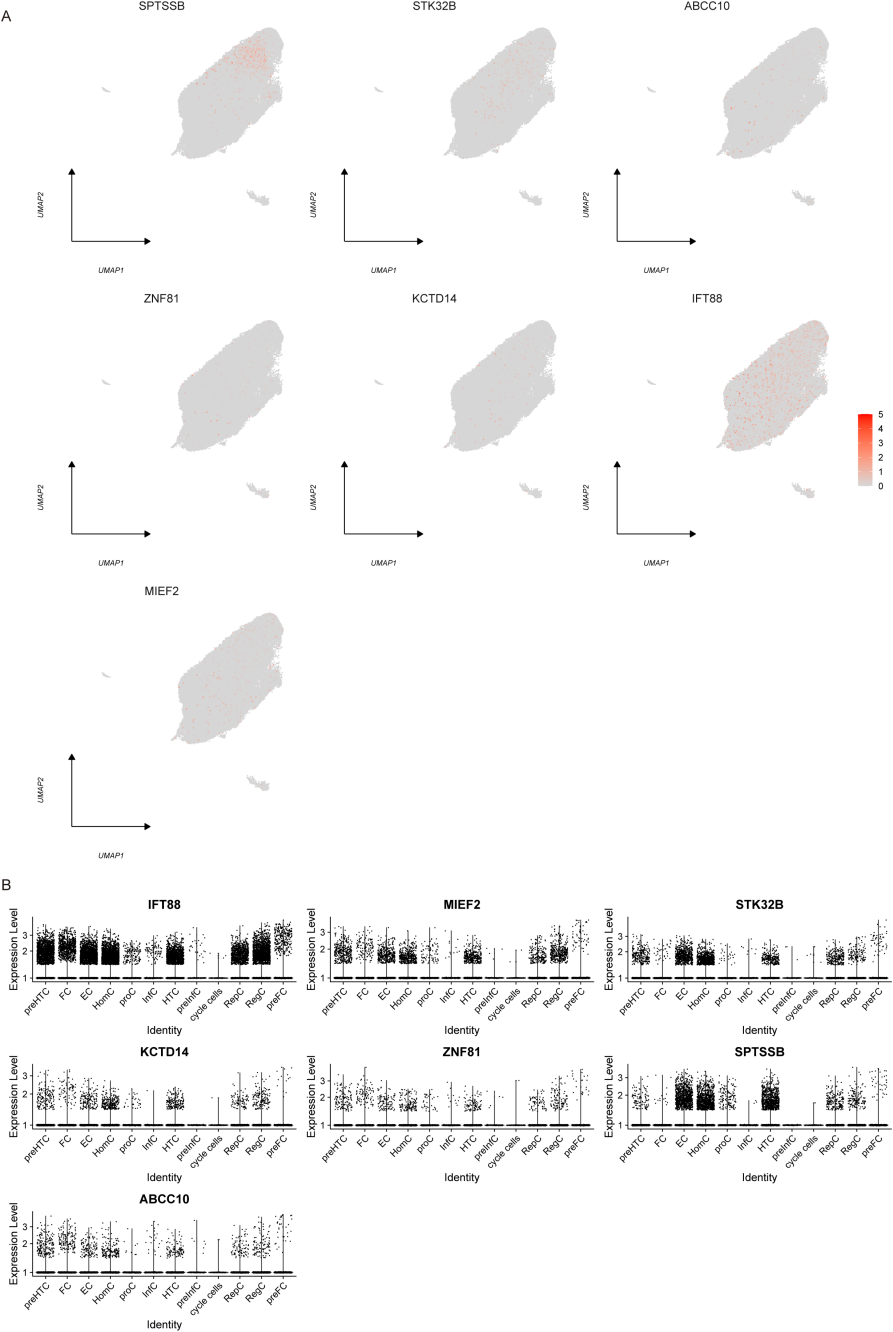
Single-cell expression. **(A)** Scatter plot showing the expression profile of key genes in single cells. **(B)** Violin plot of key gene expression in single cells.

**Figure 10:**
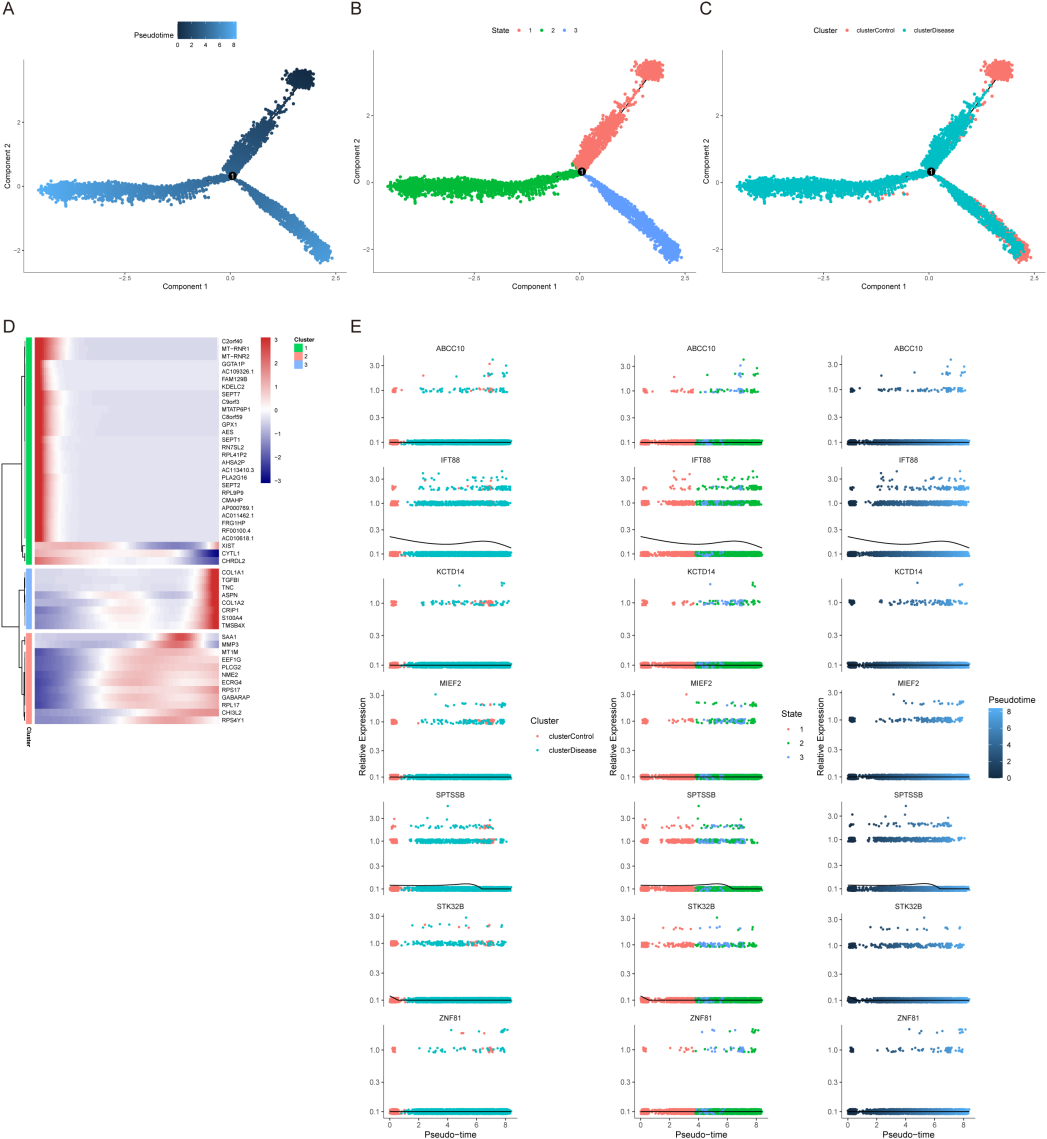
Cellular developmental trajectories. **(A–C)** Pseudotime analysis and developmental trajectories of cells. **(D)** Gene expression dynamics in each pigment cell branch. **(E)** Relationship between key gene expression and cell developmental trajectories.

We used the Dotplot, FeaturePlot and VlnPlot functions in the SeuratR package to check the expression of key genes in single cells (**Figures 9A, B**). Then we carried out a quasi-time series analysis, firstly calculating the similarity between cells and constructing the cell differentiation trajectory. Then, by visualizing the trajectory, a picture of cell differentiation trajectory constructed in pseudo-time can be generated to show the development process of cells, which can be used to study the process of cell differentiation and gene expression patterns at different time points. pseudotime value (Pseudotime is the probability calculated by monocle based on cell gene expression information, indicating the order of time), state (the block distinguished by path branches) and cell color pictures of different groups were output respectively. The results showed that the control group was mainly distributed in the early stage of cell differentiation. The disease group was mainly distributed in the late stage of cell differentiation (**Figures 10A–C**). Through the calculation, we also selected and visualized the batch of genes that changed the most along the pseudo-time difference. The horizontal coordinate is the pseudo-time value, and the vertical coordinate is the selected gene, which is divided into 3 clusters by default according to the changes of genes. We found that C2orf40, GGTA1P, FAM129B, KDELC2, SEPT7 and other genes were expressed in the early stage of locus differentiation. COL1A1, TNC, ASPN, COL1A2, S100A4 and other genes were expressed at the end of locus differentiation (**Figure 10D**). Finally, we also show changes in the expression of key genes during cell differentiation trajectories (**Figure 10E**).

## 4 Discussion

KOA is a complex and progressive disease driven by a combination of mechanical, inflammatory, and metabolic factors (33). While extensive research has focused on the inflammatory processes within KOA, the emerging role of non-apoptotic forms of cell death, specifically ferroptosis, in shaping the disease’s immune microenvironment is only now beginning to be understood (34, 35). This study provides novel insights into the ferroptosis-driven remodeling of the immune microenvironment in KOA by integrating single-cell transcriptomics with bulk RNA sequencing data. Our findings highlight the critical involvement of ferroptosis in the pathogenesis of KOA, demonstrating its influence on immune cell polarization, synovial inflammation, and extracellular matrix (ECM) degradation.

The identification of ferroptosis-active homeostasis chondrocytes (HomC) as key players in KOA pathogenesis is a major finding of this study. These cells were found to exhibit significant lipid peroxidation, a hallmark of ferroptosis, and were shown to orchestrate synovitis through fibroblast growth factor (FGF) signaling. Specifically, FGF1 and FGFR1 were identified as ligand-receptor pairs responsible for promoting ECM degradation and inflammation, amplifying the disease process. These results are consistent with previous studies suggesting that ferroptosis contributes to tissue damage and inflammation in other models of chronic disease (36, 37), but this is the first study to link it directly with chondrocyte behavior in KOA.

Our analysis using SCENIC revealed that HomC exhibit a unique regulon profile, with transcription factors such as SREBF1 and YY1 driving the activation of matrix metalloproteinases (MMPs). These findings are crucial because MMPs are central to cartilage degradation, and their dysregulation in KOA leads to the breakdown of the ECM, exacerbating joint degeneration (38, 39). This ferroptosis-mediated regulatory network offers a new perspective on how ferroptotic stress in chondrocytes may be a central mechanism that exacerbates inflammation and tissue degradation, driving KOA progression.

In addition to the direct effects on chondrocytes, we also observed alterations in immune cell profiles within the synovial microenvironment. Specifically, we found reduced resting mast cells and monocytes in KOA, which may indicate an imbalance in immune cell function. The negative correlation between ABCC10 expression and CD8+ T cells as well as M1 macrophages further underscores the interplay between ferroptosis and immune cell activity in KOA (40). This finding suggests that ferroptosis may influence the activation and polarization of immune cells, potentially contributing to the chronic inflammation observed in KOA (41). The role of mast cells, in particular, may be of interest for future research, as their involvement in tissue remodeling and immune modulation could open new avenues for immunotherapeutic interventions (42, 43).

To further explore the cellular dynamics, we employed CellChat and pseudotime trajectory analysis. These approaches revealed that ferroptosis is central to immune ecosystem remodeling in KOA, driving myeloid cell polarization, lymphocyte infiltration, and disruption of stromal-immune metabolic coupling. Our CellChat analysis prioritized the FGF signaling pathway due to its exceptional ligand-receptor intensity in ferroptosis-active homeostasis chondrocytes (HomC) (**Figures 2B, C**), a finding mechanistically supported by the established role of FGF1–FGFR1 interactions in promoting synovial fibroblast activation, MMP-13 secretion, and extracellular matrix (ECM) degradation (44–46).

Integration with pseudotime trajectory analysis demonstrated that the transition from homeostasis to a fibrotic, inflammatory phenotype in late-stage KOA coincided with elevated FGF1 expression in HomC and increased markers such as COL1A1 and TNC (39, 47). This suggests that ferroptosis-driven FGF signaling amplifies both ECM remodeling and fibrotic reprogramming, linking chondrocyte ferroptosis to synovitis and tissue fibrosis. Collectively, these findings delineate a mechanistic framework in which FGF signaling bridges ferroptosis and immune remodeling in KOA, exacerbating cartilage degradation, inflammation, and fibrosis (48, 49).Furthermore, we observed upregulation of oxidative stress pathways, particularly those regulated by NF-κB and HIF-1, implicating immune-metabolic dysregulation as a central driver of KOA progression (50).

A major strength of this study is the identification of a robust 7-gene diagnostic panel, which achieved an AUC of 1.0 in the training set and 0.78 in the validation cohort. These genes—including IFT88, MIEF2, and ABCC10—are involved in mitochondrial dynamics, inflammatory response, and iron metabolism (51–54), highlighting their potential for non-invasive early detection of KOA and for guiding future biomarker-based interventions.

In terms of therapeutic applications, our findings point to several potential targets. Ferroptosis inhibitors, which are currently under investigation in cancer therapies (55), could offer a novel approach to modulating cell death in KOA. Targeting immune cell dysfunction, particularly mast cells and macrophages, may help to restore immune balance within the joint and reduce chronic inflammation (56–58). Furthermore, inhibiting the FGF signaling axis—which has been identified as central to HomC-driven synovitis and ECM degradation—also represents a promising therapeutic strategy. Recent studies, such as that by Xu et al. (59), have further demonstrated that ferroptosis inhibitors can protect chondrocytes while reducing the expression of pro-inflammatory mediators, thereby supporting the therapeutic value of targeting ferroptosis and its associated immune-metabolic crosstalk in KOA.

While this study provides valuable insights, several limitations must be considered. First, although batch effect correction for the single-cell RNA-seq data (GSE255460) was performed using the Harmony algorithm and visually assessed by UMAP, quantitative kBET analysis showed only a 2% acceptance rate post-correction, likely due to biological variability and uneven cell type distribution. Second, the validation cohort was small (GSE246425, n=12), which may limit statistical power and generalizability, and no *a priori* sample size calculation was possible since our work is a secondary analysis of public datasets. Third, all findings are based on computational analysis without direct experimental validation. We have outlined future plans to address this, including lipid peroxidation assays, iron content measurement, and ferroptosis inhibition experiments. Future studies with larger cohorts and wet-lab experiments are needed to confirm our conclusions and further explore the underlying mechanisms.

Additionally, while the single-cell transcriptomic analysis provides high-resolution data on immune cells and chondrocytes, the spatial organization of these cells within the tissue is not fully addressed. Future studies integrating spatial transcriptomics or immunohistochemistry could provide more context on how ferroptosis and immune cells interact in the native tissue environment.

Furthermore, while we focused on ferroptosis as a key driver of immune dysfunction in KOA, other forms of non-apoptotic cell death, such as necroptosis or pyroptosis, may also contribute to the disease process. Future studies exploring these pathways could offer a more comprehensive understanding of the molecular mechanisms underlying KOA. Moreover, the role of ferroptosis in other joint diseases, such as rheumatoid arthritis, should be investigated to determine whether the findings in KOA are broadly applicable to other inflammatory conditions.

## 5 Conclusion

In summary, this study establishes ferroptosis as a key driver of immune-metabolic dysfunction in KOA, linking it to synovitis, ECM remodeling, and disease progression. By integrating single-cell transcriptomics and bulk RNA-seq, we have uncovered novel mechanisms by which ferroptosis influences immune microenvironment remodeling in KOA, providing potential biomarkers and therapeutic targets. Our findings not only enhance the understanding of KOA pathogenesis but also open new avenues for developing precision diagnostic and therapeutic strategies to better manage this debilitating disease.

## Data Availability

The original contributions presented in the study are included in the article/**Supplementary Material**. Further inquiries can be directed to the corresponding authors.
