# Supplementary material for: Single-cell transcriptome and multi-omics integration reveal ferroptosis-driven immune microenvironment remodeling in knee osteoarthritis
Source: Front Immunol. 2025 Jun 25;16:1608378. doi: 10.3389/fimmu.2025.1608378 (PMC12238886; doi:10.3389/fimmu.2025.1608378)
Supplement: Supplementary file 1 [file DataSheet1.docx]

Supplementary Material

## Supplementary Figures


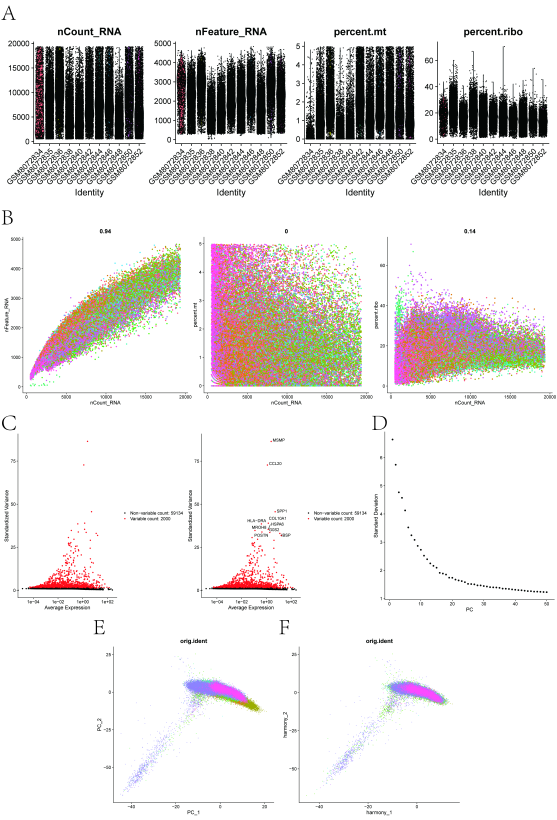


**Supplementary Figure 1.** Preprocessing of single-cell data. **(A)** Quality control of single cells, showing the number of cells, genes, and sequencing depth for each sample. **(B)** Scatter plots showing the relationship between sequencing depth and mitochondrial content (left), mitochondrial content and nCount_RNA (middle), and sequencing depth and gene numbers (right). The correlation between mitochondrial content (y-axis) and nCount_RNA (x-axis) is shown, with each point representing a single cell. **(C)** Identification of genes with significant inter-cell differences, represented by a feature variance plot. **(D)** Variance ranking plot for each principal component (PC). **(E-F)** PCA visualization and distribution of PCs, with points representing cells and colors representing samples.


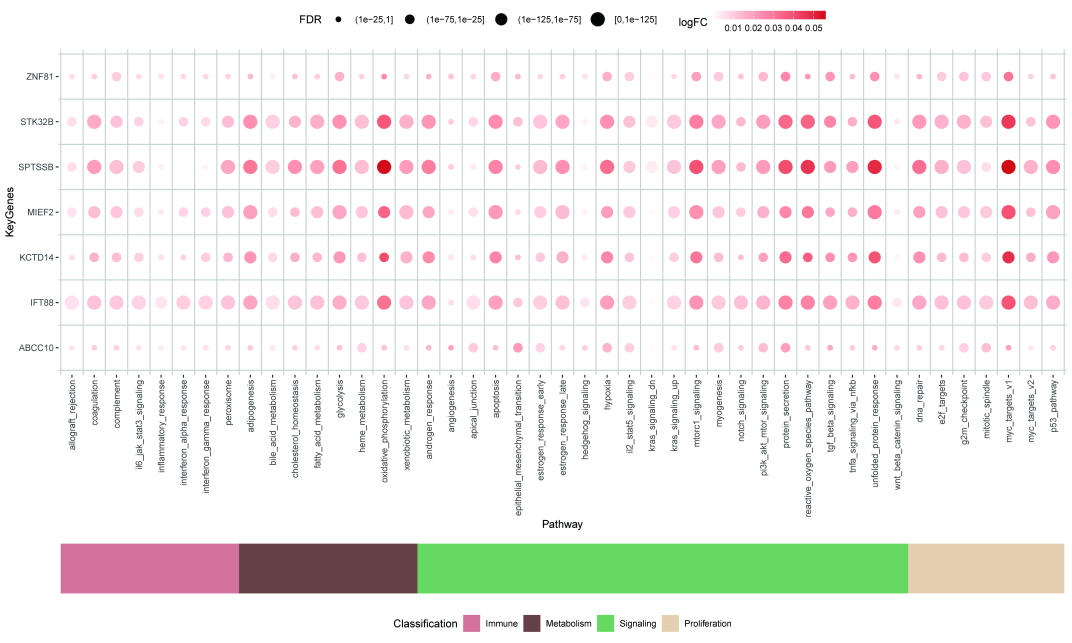


**Supplementary Figure 2.** Differences in key genes and immune metabolic pathway activities. Key genes and their differences in immune metabolic pathway activity are shown, with blue representing low expression and red representing high expression.
